# Supplementary material for: Vibronic mixing enables ultrafast energy flow in light-harvesting complex II
Source: Nat Commun. 2020 Mar 19;11:1460. doi: 10.1038/s41467-020-14970-1 (PMC7081214; doi:10.1038/s41467-020-14970-1)
Supplement: Supplementary file 1 — Supplementary Information [file 41467_2020_14970_MOESM1_ESM.pdf]

Supplementary Information

Vibronic mixing enables ultrafast energy flow in light-harvesting complex II

Arsenault et al.

Supplementary Note 1

Cresyl Violet: Results and Discussion

In order to demonstrate the role of vibrational coherences, or rather lack thereof, in 2DEV spectra, control experiments were performed on cresyl violet (CV) dissolved in deuterated methanol. CV was chosen because it is well-documented in the literature that when both two-dimensional electronic spectroscopy (2DES) and visible pump-visible probe spectroscopy have been applied to CV, strong vibrational coherences with a frequency of ~590 cm^{-1} were observed.[1–3] However, in a 2DEV experiment, performed under the same broadband excitation conditions as in previous 2DES and visible pump-visible probe experiments (Supplementary Figure 1), no

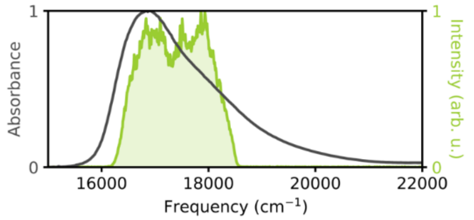

Supplementary Figure 1. Linear absorption spectrum of cresyl violet. Absorption spectrum of cresyl violet dissolved in deuterated methanol at 298 K (grey) together with laser excitation spectrum (green).

vibrational coherences were observed. In the following text, these results are presented along with a short discussion.

Representative 2DEV spectra of CV are shown in Supplementary Figure 2. Throughout the remainder of the discussion, we will apply the same analysis as in the main text to the strong

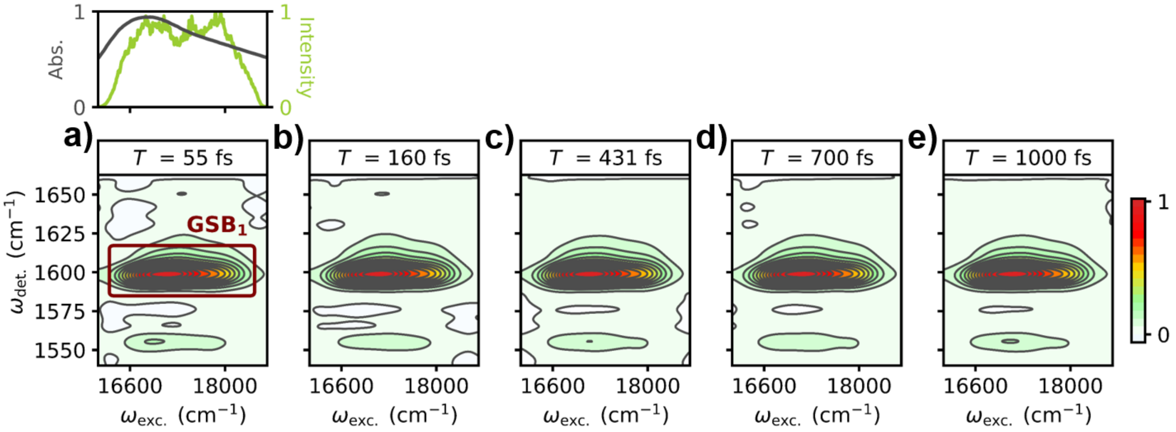

Supplementary Figure 2. 2DEV spectra of cresyl violet. a)-e) 2DEV spectra of cresyl violet at T = 55 fs, 160 fs, 431 fs, 700 fs, and 1000 fs, respectively. All spectra have been normalized to T = 0 fs. Contour levels are drawn in 6.6% intervals. The absorption spectrum and laser excitation spectrum are shown above a) and the main feature of interest, GSB$_1$, has been labeled in a).

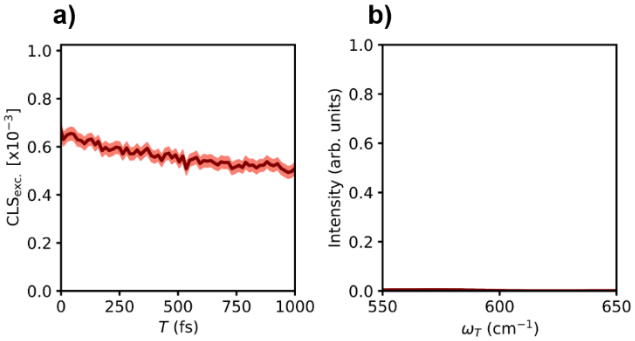

Supplementary Figure 3. Center line slope (CLS) dynamics of cresyl violet. a) $CLS_{exc.}$ dynamics of GSB_1 (dark red line) along with the standard error from the linear fits used to calculate the $CLS_{exc.}$ (light red shaded region). b) Power spectrum of the $CLS_{exc.}$ dynamics of GSB_1 centered around the region of interest, ~590 cm^{-1}. In both a) and b), there is no evidence for wavepacket dynamics.

ground state bleach (GSB) feature labeled as GSB_1 in Supplementary Figure 2. The results of the center line slope (CLS) analysis for GSB_1 along the excitation axis are shown in Supplementary Figure 3a. It is immediately apparent that no oscillatory dynamics are present. To make this even more obvious, the power spectrum of the CLS is shown in Supplementary Figure 3b—there is absolutely no evidence for wavepacket dynamics in the CLS. To complete the analysis, we show slices through GSB_1 taken along the excitation axis and plotted as a function of waiting time, *T*, in Supplementary Figure 4 and we show the power spectrum of GSB_1 plotted along with the noise

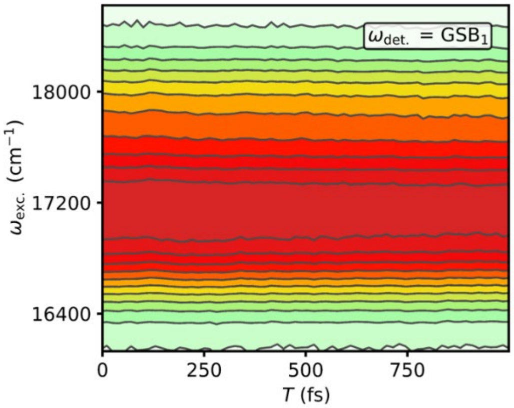

Supplementary Figure 4. Spectral dynamics of cresyl violet along the excitation axis at a fixed detection frequency. To highlight the lack of oscillatory intensity dynamics in the 2DEV spectra of cresyl violet, slices were taken through GSB_1 and plotted as a function of waiting time, *T*. The colormap used here is identical to that of Supplementary Figure 4.

floor in Supplementary Figure 5. Once again, no oscillatory dynamics were observed, which demonstrates that the 2DEV spectra of CV do not exhibit the vibrational coherences that one would expect to see based on previous 2DES or visible pump-visible probe experiments. The reason that these vibrational coherences are not observable in this 2DEV experiment is because the probed

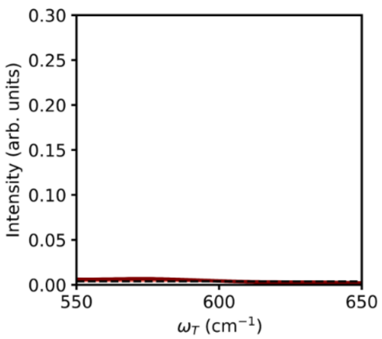

Supplementary Figure 5. Power spectrum of GSB_1. Average power spectrum of GSB_1 (red) centered around the region of interest, ~590 cm^{-1}, versus the average noise floor (black, dashed) calculated by taking the average power spectrum of the experimental noise. No wavepacket dynamics are evident.

higher frequency vibrational modes are not anharmonically coupled to the lower frequency modes[4] that are responsible for the appearance of the vibrational coherences in previous work. The result is that the probed modes are completely ignorant of the wavepacket dynamics. Therefore, these dynamics are absent from the CLSs and peak amplitudes. This conclusion has important implications for 2DEV spectroscopy—in the case where the probed vibrational modes are not anharmonically coupled to those that would be responsible for the appearance of vibrational coherences in a 2DES experiment, any observed oscillations in the CLS or peak amplitude in a 2DEV experiment will *not* be of purely vibrational origin, but rather must be electronic or vibronic in origin. In terms of the current study, the vibrational modes of chlorophyll pigments in the probed region (1525-1715 cm^{-1}) are not anharmonically coupled to lower frequency modes (i.e. the frequency region of the observed beats in the current study).[5] Therefore, the observed beats can *not* be vibrational in origin.

Cresyl Violet: Methods

The same 2DEV experimental setup described in the main text and elsewhere[6] was used for this experiment, however, the excitation and probe frequencies were adjusted accordingly. The visible excitation spectrum was centered at 17390 cm^{-1} and spanned 16129~18518 cm^{-1}, while the IR probe spectrum was centered at 1587 cm^{-1}. The excitation energy was set at 250 nJ and the pump pulse duration was ~8 fs.

Supplementary Note 2

Theoretical Modelling

The model presented in the main text features two electronically coupled monomers, each with one electronic degree of freedom (DoF) and one Franck-Condon active vibrational mode. The model parameters (labeled in Supplementary Figure 6) were chosen to be similar to those expected for LHCII and are as follows: $\epsilon_\beta - \epsilon_\alpha = 100$ cm^{-1}, $J = 100$ cm^{-1}, $\omega_{g,\alpha} = 1650$ cm^{-1}, $\omega_{e,\alpha} = 1560$ cm^{-1}, $\omega_{g,\beta} = 1660$ cm^{-1}, $\omega_{e,\beta} = 1550$ cm^{-1}, and the Huang-Rhys factor, S, was set to 0.005 for

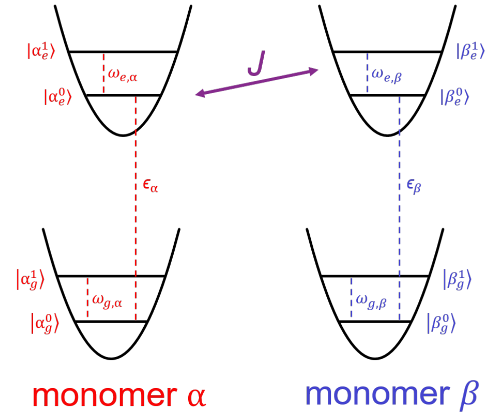

Supplementary Figure 6. Schematic of heterodimer model in the site basis. In the model, the strength of electronic coupling between the electronically excited states of monomers α and β is given by J. Labeled are the various model parameters (energies of vibrational and electronic degrees of freedom for each monomer) and the states for each monomer.

both vibrational modes.[7–9] As in previous work[10,11], the site basis is spanned by the nine states: $|\alpha_g^0\beta_g^0\rangle$, $|\alpha_g^1\beta_g^0\rangle$, $|\alpha_g^0\beta_g^1\rangle$, $|\alpha_e^0\beta_g^0\rangle$, $|\alpha_e^0\beta_g^1\rangle$, $|\alpha_e^1\beta_g^0\rangle$, $|\alpha_g^0\beta_e^0\rangle$, $|\alpha_g^0\beta_e^1\rangle$, and $|\alpha_g^1\beta_e^0\rangle$, where the subscript on α or β indicates whether or not the electronic DoF is excited (e for excited state or g for ground state) and the superscript indicates whether or not the vibrational DoF on monomer α or β is excited (1 for excited state or 0 for ground state). In the site basis, the electronic transition dipole matrix elements were set to $\frac{\mu_\alpha}{\mu_\beta} = -5$, in order to recover a linear absorption spectrum with reasonable qualitative agreement to LHCII in terms of the intensities of the two main Q_y bands, and the vibrational transition dipole matrix elements were all set to unity.

The simulated 2DEV spectra (composed of the rephasing and nonrephasing excited state absorption and ground states bleach pathways[10]) were calculated using the Full Redfield quantum master equation.[12] The spectral density was taken to be of Drude-Lorentz form[13], where the

reorganization energy, λ, was set to 35 cm^{-1} and the cutoff frequency, γ, was set to ~106 cm^{-1} ($\gamma^{-1} = 50$ fs). These bath parameters were chosen as they are reasonable for pigment-protein complexes.[14,15] For all calculations, the temperature was set at 150 K, rather than 77 K, in order to ease computational demand. As the vibrational modes in this model were assumed to be localized spectators of the dynamics and were not populated until the third light-matter interaction, an electronic bath that induced energy fluctuations and population relaxation was used during the initial coherence time, t_1, and the waiting time, T, while a vibrational bath that only induced vibrational relaxation was used during the last coherence time, t_3 (note: this would not be the proper treatment in the case where there was substantial electronic-vibrational mixing[16], however, the separation of the bath into discrete electronic and vibrational portions is valid for the parameter regime inhabited by in this model, which is essentially that of "D1" in Ref. 17). The absorptive 2DEV spectra presented in the text were recovered by Fourier transforming along t_1 and t_3 and combining the total rephasing and nonrephasing pathways. The spectra were calculated as a function of T from 0 to 708 fs in 12 fs steps.

In order to highlight the sensitivity of 2DEV to electronic coupling, the 2DEV spectrum for $J = 0$ cm^{-1} (all other model parameters identical) is shown in Supplementary Figure 7. Through a comparison of Figure 3b-c (in main text) to Supplementary Figure 7, it is evident that the two excited state absorption quartets arise as a result of electronic coupling.

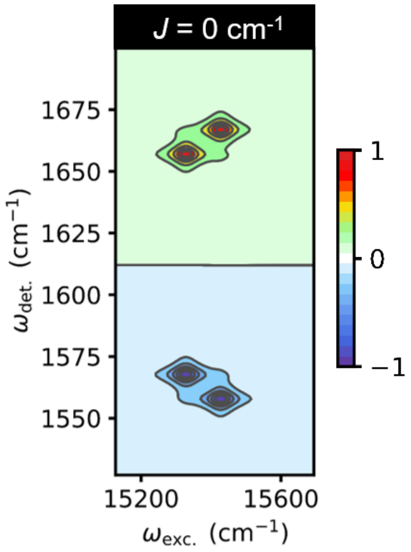

Supplementary Figure 7. Theoretical 2DEV spectrum for the model with no electronic coupling. When $J = 0$ cm^{-1}, the excited state absorption (ESA) quartet structure vanishes, leaving only one ESA feature on each non-interacting monomer. Positive features indicate ground state bleaches and negative features indicate ESAs.

Supplementary Note 3

LHCII: Additional Experimental Details

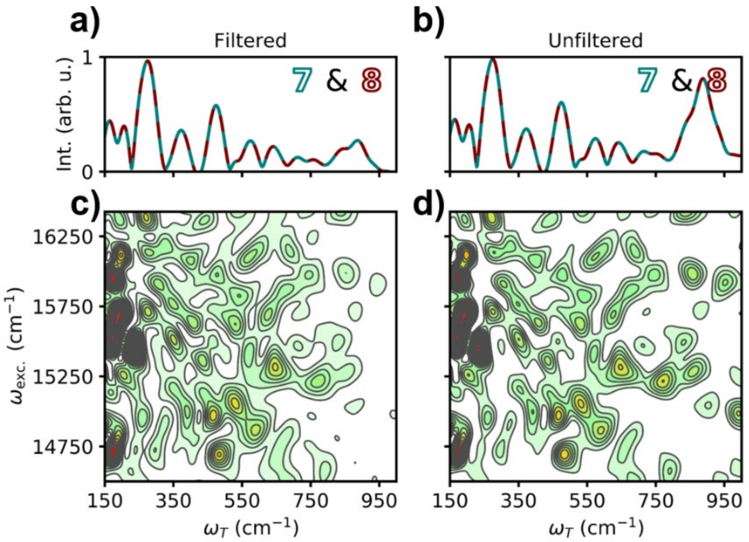

Supplementary Figure 8. Filtered versus unfiltered center line slope cross-power spectrum and beat frequency map along the excitation axis. a)-b) Filtered (as described in the Methods section) and unfiltered cross power spectrum of the center line slope dynamics of excited state absorptions seven and eight. c)-d) Filtered (again, as described in the Methods section) and unfiltered beat frequency map. Only peaks that survive the noise floor were plotted in c) and d) such that contour levels are drawn in 4% intervals starting from the top of the noise floor. The colormap indicates peak intensity, where intensity is shown to increase from green to red.

As mentioned in the Methods section, the experimental data was subjected to a Savitzky-Golay filter[18] for presentation in the main text, in order to emphasize the lower frequency oscillatory signals under discussion. In Supplementary Figure 8, we compare the both the filtered and unfiltered versions of the cross-power spectrum and beat frequency map presented in Figure 5 of the main text. Upon comparison, it is evident that filtering leaves the oscillatory signals under discussion unaltered, rather, as intended, filtering only deemphasizes higher frequency signals.

A more explicit comparison of the beat frequencies versus the noise floor is shown in Supplementary Figure 9. The noise floor was calculated by taking the average power spectrum of the experimental noise.

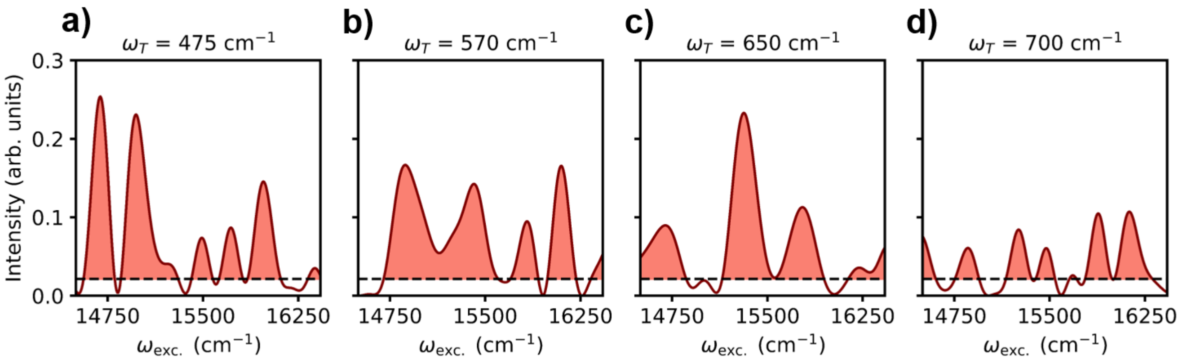

Supplementary Figure 9. Selected beat frequencies versus the noise floor. a)-d) Selected beat frequencies of 475 cm^{-1}, 570 cm^{-1}, 650 cm^{-1}, and 700 cm^{-1} plotted as a function of excitation frequency shown against the average noise floor (black, dashed). Features that survive the noise floor are shaded in red.

The linear absorption spectrum of LHCII at 77 K along with the laser excitation spectrum is presented in Supplementary Figure 10.

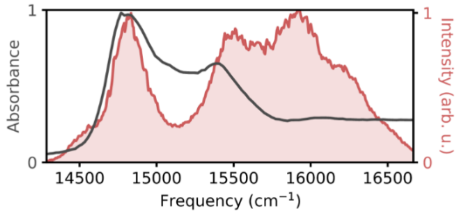

Supplementary Figure 10. Linear absorption spectrum of LHCII AT 77 K. Absorption spectrum of LHCII at 77 K (grey) together with laser excitation spectrum (red).

Supplementary References

1. Turner, D. B., Wilk, K. E., Curmi, P. M. G. & Scholes, G. D. Comparison of electronic and vibrational coherence measured by two-dimensional electronic spectroscopy. *J. Phys. Chem. Lett.* **2**, 1904–1911 (2011).

2. Bizimana, L. A., Brazard, J., Carbery, W. P., Gellen, T. & Turner, D. B. Resolving molecular vibronic structure using high-sensitivity two-dimensional electronic spectroscopy. *J. Chem. Phys.* **143**, 164203 (2015).

3. Rafiq, S. & Scholes, G. D. Slow intramolecular vibrational relaxation leads to long-lived excited-state wavepackets. *J. Phys. Chem. A* **120**, 6792–6799 (2016).

4. Vogel, E., Gbureck, A. & Kiefer, W. Vibrational spectroscopic studies on the dyes cresyl

violet and coumarin 152. *J. Mol. Struct.* **550–551**, 177–190 (2000).

5. Barone, V. *et al.* Toward anharmonic computations of vibrational spectra for larger molecular systems. *Int. J. Quantum Chem.* **112**, 2185–2200 (2012).

6. Oliver, T. A. A., Lewis, N. H. C. & Fleming, G. R. Correlating the motion of electrons and nuclei with two-dimensional electronic-vibrational spectroscopy. *Proc. Natl. Acad. Sci.* **111**, 10061–10066 (2014).

7. Novoderezhkin, V. I., Palacios, M. A., Van Amerongen, H. & Van Grondelle, R. Energy-transfer dynamics in the LHCII complex of higher plants: modified Redfield approach. *J. Phys. Chem. B* **108**, 10363–10375 (2004).

8. Novoderezhkin, V. I., Palacios, M. A., Van Amerongen, H. & Van Grondelle, R. Excitation dynamics in the LHCII complex of higher plants: modeling based on the 2.72 Å crystal structure. *J. Phys. Chem. B* **109**, 10493–10504 (2005).

9. Lewis, N. H. C. *et al.* Observation of electronic excitation transfer through light harvesting complex II using two-dimensional electronic-vibrational spectroscopy. *J. Phys. Chem. Lett.* **7**, 4197–4206 (2016).

10. Bhattacharyya, P. & Fleming, G. R. Two-dimensional electronic-vibrational spectroscopy of coupled molecular complexes: a near-analytical approach. *J. Phys. Chem. Lett.* **10**, 2081–2089 (2019).

11. Polyutov, S., Kühn, O. & Pullerits, T. Exciton-vibrational coupling in molecular aggregates: electronic versus vibronic dimer. *Chem. Phys.* **394**, 21–28 (2012).

12. Ishizaki, A. & Fleming, G. R. On the adequacy of the Redfield equation and related approaches to the study of quantum dynamics in electronic energy transfer. *J. Chem. Phys.* **130**, (2009).

13. Mukamel, S. *Principles of Nonlinear Optical Spectroscopy*. (Oxford Univeristy Pres, 1995).

14. Cheng, Y.-C. & Fleming, G. R. Dynamics of light harvesting in photosynthesis. *Annu. Rev. Phys. Chem.* **60**, 241–262 (2009).

15. Ishizaki, A. & Fleming, G. R. Quantum coherence in photosynthetic light harvesting. *Annu.*

Rev. Condens. Matter Phys. **3**, 333–361 (2012).

16. Yeh, S.-H., Hoehn, R. D., Allodi, M. A., Engel, G. S. & Kais, S. Elucidation of near-resonance vibronic coherence lifetimes by nonadiabatic electronic-vibrational state character mixing. *Proc. Natl. Acad. Sci.* (2018). doi:10.1073/pnas.1701390115

17. Butkus, V., Valkunas, L. & Abramavicius, D. Vibronic phenomena and exciton-vibrational interference in two-dimensional spectra of molecular aggregates. *J. Chem. Phys.* **140**, 034306 (2014).

18. Savitzky, A. & Golay, M. J. E. Smoothing and differentiation of data by simplified least squares procedures. *Anal. Chem.* **36**, 1627–1639 (1964).
